# 928. Concordance with Institutional Guidelines for Letermovir Cytomegalovirus Infection Prophylaxis in Allogeneic Hematopoietic Stem Cell Transplant Recipients

**DOI:** 10.1093/ofid/ofad500.973

**Published:** 2023-11-27

**Authors:** Alex Peterson-Weber, Nicholas Palisano, Carolyn D Alonso

**Affiliations:** Beth Israel Deaconess Medical Center, Boston, Massachusetts; Beth Israel Deaconess Medical Center, Boston, Massachusetts; Beth Israel Deaconess Medical Center, Boston, Massachusetts

## Abstract

**Background:**

Cytomegalovirus (CMV) is an opportunistic infection in immunocompromised patients associated with significant morbidity and mortality. Letermovir (LET) is used for prophylaxis for CMV-seropositive allogeneic hematopoietic stem cell transplant (HSCT) recipients. An institutional guideline was developed to provide guidance on the appropriate use of letermovir. The purpose of this study was to determine overall concordance with letermovir institutional guidelines and to identify areas for quality improvement.

**Methods:**

Our evaluation included adult hospitalized patients admitted from November 2019 to July 2022 for allogeneic HSCT who received LET, identified via medication administration records. The primary outcome was concordance with the institutional guideline criteria for use, as outlined in Figure 1. Secondary outcomes included incidence of breakthrough CMV viremia, use of concomitant HSV prophylaxis, and appropriate management of drug interactions (Figure 1).

**Figure:**
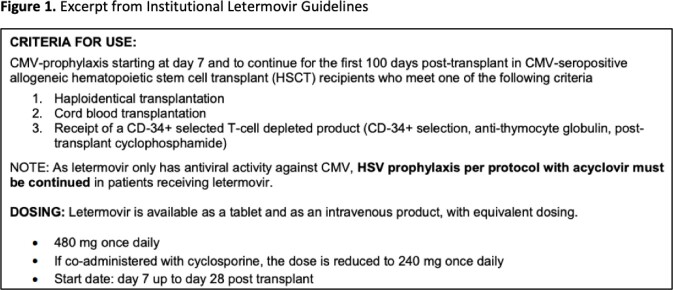

**Results:**

We identified 41 patients who received LET during the study period. After excluding patients who received a solid organ transplant, 32 patients were included in the study. The median time to LET initiation post-transplant was 7 days and the median time to discontinuation was 105 days post-transplant. Overall, 9 patients (28%) were fully concordant with our guidelines. Of the remaining patients, reasons for discordance are summarized in Table 1, mainly driven by continuation of LET >110 days post-transplant (n=12, 37%). Three (9%) patients received LET despite CMV recipient negative serostatus (CMV R-). Breakthrough CMV viremia occurred in 3 patients (9%). All patients received concomitant HSV prophylaxis and had drug interactions managed appropriately.

**Figure:**
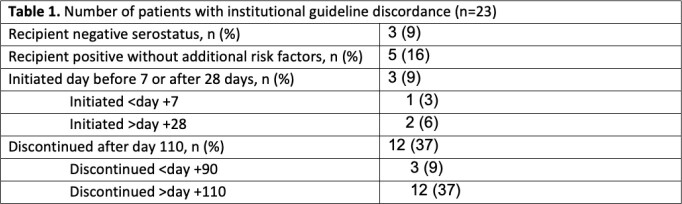

**Conclusion:**

Our evaluation identified a lower than expected rate of letermovir guideline concordance, primarily related to the recommended duration of drug therapy. We found that our guideline has supported providers to ensure that concomitant HSV prophylaxis is given and drug interactions are managed appropriately. A further investigation into the use of letermovir in CMV R- subjects and a cost analysis is planned to guide future institutional guideline updates.

**Disclosures:**

**Carolyn D. Alonso, MD**, Academy for Continued Healthcare Learning: Honoraria|AiCuris: Advisor/Consultant|American Society of Healthcare Pharmacists: Honoraria|Cidara Therapeutics: Advisor/Consultant|Clinical Care Options: Honoraria|Merck: Advisor/Consultant|Merck: Grant/Research Support

